# The Relation of the Home Literacy Environment to White Matter Pathways Associated with Language in Children 5–8 Years Old

**DOI:** 10.1162/NOL.a.272

**Published:** 2026-07-10

**Authors:** Alisha B. Compton, Avantika Mathur, James R. Booth

**Affiliations:** Department of Psychology and Human Development, Vanderbilt University, Nashville, TN, USA

**Keywords:** diffusion imaging, home literacy environment, phonology, semantics, shared reading, white matter

## Abstract

Prior work suggests relations between the home literacy environment (HLE) and white matter in children less than 7 years of age, but studies have not yet examined the relation of HLE to white matter pathways in older children. A study on young children investigated two dorsal tracts (arcuate fasciculus [AF] and superior longitudinal fasciculus [SLF]) and one ventral tract (inferior longitudinal fasciculus [ILF]), but not the inferior fronto-occipital fasciculus (IFOF). Moreover, this study did not use a method to examine specific relations with phonological or semantic mechanisms. The present study examined the relation of HLE to these four white matter pathways in a group of children 5–6 years old (*N* = 79, *M*_age_ = 5.9) and a group of children 7–8 years old (*N* = 107, *M*_age_ = 7.6). Participants completed diffusion weighted imaging scans and standardized assessments of phonology and semantics. Preregistered node-wise regression analyses tested relations of HLE to fractional anisotropy (FA). Relations were examined across 100 nodes and in the 30 nodes most associated with phonological awareness in the left AF and left SLF or semantic knowledge in the left IFOF and left ILF. Results only suggest a relation of parent-to-child reading and FA in the left IFOF for the group of children 5–6 years old. We suggest this relation may be related to the importance of picture naming in early shared reading of books. Given the limited relations demonstrated in the current preregistered study with relatively large samples, coupled with inconsistent findings in the literature, future research is needed to investigate robust relations of HLE to white matter.

## INTRODUCTION

Language and reading are skills that can influence academic achievement, career success, and overall well-being (Gross, 2006). Children vary greatly in these skills; however, we do not fully understand what underlies this variability. As children gain language and reading skills, researchers have examined how the brain changes in terms of its structure. Fractional anisotropy (FA) is a metric of diffusion of water molecules that has been used to study white matter tracts. FA changes and generally shows increases overall across most major white matter tracts as children develop, which are due to many physiological processes, including microscopic factors such as increases in myelination and macroscopic factors such as directional coherence of axons (Lebel et al., 2008). Yeatman, Dougherty, Myall, et al. (2012) built on these findings to show that FA increases between late childhood and early adolescence are localized to specific subregions of the tracts. The authors suggest that these increases in FA in specific parts of the tract may reflect developmental changes within distinct populations of axons (Yeatman, Dougherty, Myall, et al., 2012). Studies have also found an increase in FA in specific regions and groups of white matter tracts as children acquire relevant skills (Vandermosten et al., 2012). In a review on diffusion tensor imaging (DTI) and dyslexia, Vandermosten et al. (2012) found that lower FA in temporoparietal and frontal areas correlates with lower reading skills.

Two critical components of language are phonological and semantic processing, which serve as foundations for reading acquisition (Frost et al., 2005; Wang et al., 2020). The arcuate fasciculus (AF) has been associated with phonology in adults (Schwartz et al., 2012) and children (Vandermosten et al., 2015; Yeatman et al., 2011). In contrast, the inferior fronto-occipital fasciculus (IFOF) has been associated with semantics in adults (Mirman et al., 2015) and children (Vanderauwera et al., 2018). The superior longitudinal fasciculus (SLF) and inferior longitudinal fasciculus (ILF) have also been suggested to be part of the pathways for phonology and semantics, respectively, in adults (Harvey & Schnur, 2015), children (Travis et al., 2017), and across both age groups (Vandermosten et al., 2012), though there is less evidence supporting these specific relations. A review paper on white matter tracts and language supported the relation of AF and SLF to phonology and IFOF and ILF to semantics (Shekari & Nozari, 2023). Another large-scale study in children younger than 9 years old did not find significant white matter differences in children with reading disability compared to those without, or significant relations between white matter and reading skill (Meisler & Gabrieli, 2022). However, this study did not examine specific relations with phonological awareness or semantic skill.

In dual-stream frameworks, dorsal tracts (including AF/SLF) support phonological transformations and sensorimotor mappings for speech, as well as aspects of controlled retrieval and sequencing, whereas ventral pathways (including IFOF/ILF) support semantic–lexical mapping and combinatorial meaning (Friederici & Gierhan, 2013; Hickok & Poeppel, 2007; Saur et al., 2008). Additionally, literature on electrical stimulation of white matter tracts during awake surgery suggests that the IFOF contributes to functions including language and reading (Giampiccolo et al., 2025), and that the AF contributes to language (Giampiccolo & Duffau, 2022) and specifically to phonology (Zemmoura et al., 2015).

The home literacy environment (HLE), often defined as reading-related resources and experiences in a child’s home, has been found to be related to language and reading skill (Sénéchal & LeFevre, 2014). The type of books read and the nature of shared reading practices can vary greatly (Flack et al., 2018; Maddigan et al., 2005; Riordan et al., 2018; Stadler & McEvoy, 2003). Prior literature has found specific relations of HLE to phonological awareness (Niklas & Schneider, 2013) and vocabulary (semantic) knowledge (Sénéchal & LeFevre, 2014) in children followed longitudinally from kindergarten through first or second grade. Brain imaging studies using functional magnetic resonance imaging (fMRI) have further underscored the relation of HLE to specific language skills. Prior fMRI studies have found associations between HLE and functional activation in areas associated with phonology in younger children (Powers et al., 2016) and semantics in older children (Girard et al., 2021). Furthermore, one fMRI study investigated the relation of HLE to both semantic and phonological tasks (Compton et al., 2026). The authors examined a group of children 5–6 years old and a group of children 7–8 years old and found a relation between family-to-child reading and phonological processing in younger children and semantic processing in older children (Compton et al., 2026). The authors note that young children are still acquiring foundational skills related to phonological awareness while older children, having mastered these foundational skills, may be more focused on vocabulary growth (Ehri, 2020; Sénéchal & LeFevre, 2014). They also suggest that there may be age-related differences in the books and types of shared reading that takes place. For example, younger children often enjoy rhyming and alphabet books, whereas older children may enjoy stories with more complex plot lines (Maddigan et al., 2005). In other words, HLE not only provides opportunities for language exposure, both auditorily and in print, as well as parent–child interaction, but can also provide specific exposure to or practice with phonological skills and vocabulary depending on the type of shared reading that takes place.

DTI studies have also found relations between HLE and white matter pathways in the brain for children in infancy, preschool, and kindergarten (Davison et al., 2023; Hutton et al., 2020; Turesky et al., 2022). One longitudinal DTI study examined the relation of shared reading and number of books in the home to white matter pathways in a group of children ages 4.8–6.2 years old in kindergarten. They also examined associations of white matter pathways with language and reading skill at the initial timepoint and at follow-up when those children were 7.5–9.6 years old in second grade (Davison et al., 2023). This study found a positive relation between shared reading time and the FA of the left AF as well as left lateralization of the SLF at the initial timepoint, when children were in kindergarten. Furthermore, they found that the relation between HLE and reading skill in second grade was mediated by left lateralization of the SLF at kindergarten. No significant relations were observed with the ILF, which was the only other tract that the study examined. They did not examine relations of HLE with the FA of IFOF.

Some literature has investigated the development of dorsal tracts, including the AF and SLF, compared to ventral tracts, including the IFOF and ILF. For example, one study examined 159 children 4–11 years old, who were scanned twice and found similar annual mean percent FA change in the left IFOF, left ILF, and left SLF (Krogsrud et al., 2016). However, the authors also observed nonlinear trajectories of the left IFOF and left ILF, suggesting a deceleration of change with age. Additional studies have examined tract changes from infancy through late adulthood (Brauer et al., 2013; Lebel et al., 2012) and similarly suggest a more protracted development of the SLF and AF compared to the IFOF and ILF, with FA across tracts peaking in adulthood. In addition to examining average FA across a whole tract, some studies have examined FA development across different portions of the tract (Yeatman, Dougherty, Myall, et al., 2012) and found that in older children, 10–14 years old, FA changes persist in portions of the AF and IFOF even as maturation occurs. Taken together, the literature suggests that FA of all four of these tracts does change and generally increase throughout childhood, though the dorsal tracts show a more delayed maturational trajectory than the ventral tracts.

Some literature suggests that because the dorsal tracts (AF and SLF) may undergo a relatively more protracted development, they may be particularly sensitive to environmental influences throughout early and middle childhood (Romeo et al., 2018). Consistent with this protracted development, Romeo et al. (2018) investigated the relation of language exposure to FA in children 4–6 years old and only found relations with dorsal language tracts (left AF and left SLF). More specifically, audio recordings were used to measure specific components of home language exposure (e.g., conversational turns). Primary analyses found relations between these metrics and the left AF and left SLF, such that the anterior and posterior end of dorsal tracts showed strong correlations between conversational turns and FA. Post hoc analyses of the whole brain did not find evidence of relations with any other white matter tracts (Romeo et al., 2018). In older children, 7–15 years old, changes in FA in dorsal (left AF) and ventral (left ILF) tracts have been shown to be related to reading skill (Yeatman, Dougherty, Ben-Shachar, et al., 2012). Studies on socioeconomic status (SES), educational attainment, and white matter development have also found relations between environmental factors and dorsal (e.g., educational opportunity and FA of left AF; Roy et al., 2024) and ventral tracts (e.g., parent education and FA of bilateral IFOF; Roy et al., 2024). Although prior work suggests relations between HLE and white matter in younger children (4.8–6.2; Davison et al., 2023), studies have not yet examined the relation of HLE to white matter pathways in older children. Moreover, relations of HLE to specific portions of the tracts associated with phonology and semantics have not been investigated.

The present study aimed to build on this prior literature by examining the relation of family-to-child reading to white matter pathways associated with language in a group of young children 5–6 years old and a group of older children 7–8 years old. Given the established relations of specific components of language to white matter tracts, we focused our investigation on pathways that support distinct aspects of language processing: dorsal pathways (left AF and left SLF) that primarily subserve phonological processing and ventral pathways (left IFOF and left ILF) that predominantly support semantic processing. We also aimed to see if there are relations between family-to-child reading and FA of the portions of the left AF and left SLF most associated with phonological awareness and if there are relations between family-to-child reading and FA of the portions of the left IFOF and left ILF most associated with semantic knowledge.

We anticipated that HLE would be related to FA of these pathways and that HLE may be strongly related to phonology-associated tracts in younger children and semantic-associated tracts in older children. Some researchers have begun to examine the tract at a node-wise level, dividing the tract into equidistant sections called nodes, given that white matter properties have been shown to vary across different portions of the tract (Yeatman, Dougherty, Myall, et al., 2012). We tested these hypotheses by identifying 30 nodes most correlated with phonological awareness in dorsal pathways and semantic knowledge in ventral pathways. In these analyses, those 30 nodes represent discrete portions along each white matter tract where FA values are most correlated with phonological awareness or semantic knowledge, allowing for spatially specific analysis of tract microstructure. Our preregistered primary planned analyses calculated partial correlations (1) examining the relation of HLE to the FA in the 30 nodes most strongly associated with phonology in the left AF and left SLF for each age group and (2) examining the relation of HLE to the FA in the 30 nodes most strongly associated with semantics in the left IFOF and left ILF for each age group. We controlled for SES and age in partial correlations. In secondary planned analyses, we also calculated these partial correlations for the entirety of each of these tracts regardless of their relation with phonology and semantics, which amounted to 100 nodes per tract.

## METHODS

### Materials and Methods

This study uses part of an existing data set that is shared on OpenNeuro.org. Detailed information about the data set can be found in the data descriptor by Wang et al. (2022). All the experimental procedures were approved by the institutional review board (IRB) of The University of Texas at Austin (IRB Protocol No. 2014-07-0018). Informed consent was collected from participants’ parents or guardians, and assent was collected from children before participation in the study. The research questions, hypotheses, and analytical plan were preregistered through the Open Science Framework prior to the start of data analyses (see https://osf.io/9mhr8/). Two deviations from the preregistration were made, which are detailed in the Deviations From Preregistration section. The list of participants and runs used in the current study, as well as the code used to analyze the data, were shared on https://github.com/comptoab/HLE_PhonSem_WhiteMatter.

### Participants

#### Number of participants

Children who had data for their diffusion weighted imaging (DWI) scan and relevant survey data were screened for inclusionary criteria, which are detailed below, along with descriptions of relevant surveys and assessments. Two separate age groups were assessed for inclusionary criteria; given that there is a gap in age between the groups, the data were not treated as one continuous group. A Wilcoxon rank-sum test was used to examine if the groups significantly differed on age in months, given that the groups varied in size and displayed non-normal distributions for age in months (see the HLE Partial Correlation Analyses—Primary Planned Analyses section). The groups significantly differ in age (*p* < 2.2e−16), supporting the decision to analyze each group separately.

A total of 86 children 5–6 years old had DWI, HLE, and SES measures. After screening for additional inclusionary criteria, 79 children were included in analysis for the younger age group. More specifically, in this group, two children had incomplete DWI scans, one child was excluded based on handedness, one child was missing standardized nonverbal IQ data, one child displayed variations from Mainstream American English (MAE), and one child’s parent noted that their child had a psychiatric disorder. After removing these individuals from the sample, one child’s phonology standardized score was more than 3 standard deviations from the mean, so that child was also not included in analysis, bringing the total sample to 79 children 5–6 years old.

For the older group, a total of 122 children 7–8 years old had DWI, HLE, and SES measures and thus were also screened in this study for the older cohort. After screening for additional inclusionary criteria, 107 children were included in analysis for the older age group. More specifically, in this group, one individual was excluded because not enough fibers could be identified to define their DTI tracts, two children were excluded based on handedness, one child was missing standardized phonology data, eight children displayed variations from MAE, one child’s parent noted that their child had a psychiatric disorder, and one child’s parent noted that their child had a learning disorder. After removing these individuals from the sample, one child’s semantics standardized score was more than 3 standard deviations from the mean, so that child was also not included in the analysis, bringing the total sample to 107 children 7–8 years old.

#### Inclusionary criteria details

Participants needed to have relevant brain data (DWI scan), survey data (HLE and SES information), and standardized assessment scores (phonology, semantics, and nonverbal IQ). Additionally, the following four inclusionary criteria were implemented: The first was (a) right-handedness, defined as completing at least three of the five handedness tasks with the right hand (write, draw, pick up, open, and throw). The second was (b) speaking MAE as measured by the Diagnostic Evaluation of Language Variation (Seymour et al., 2003), Part 1 Language Variation Status subtest. Children’s scores were categorized as MAE based on the manual for this test for each of the age groups: seven of 15 responses for 5-year-olds, eight of 15 responses for 6-year-olds, nine of 15 responses for 7-year-olds, and 11 of 15 responses for 8-year-olds. The third was (c) no neurological, psychiatric, or learning disorders, according to the Developmental History Questionnaire. Lastly, (d) typical hearing and typical or corrected-to-normal vision, as reported by a parent or guardian.

#### Survey measures

Parents or guardians of the younger and older children were asked to complete surveys, including an eligibility survey and a Developmental History Questionnaire. For both groups, SES measured as maternal education (highest degree completed) was extracted from the Developmental History Questionnaire. For the younger group, HLE scores were extracted from the Developmental History Questionnaire, measuring family-to-child reading: the amount of time a child is read to by a family member each day (“How long per day is your child read to by an adult?”: 0–15 min, 15–30 min, 30–60 min, more than 1 hr per day, and more than 2 hr per day). For the older group, a Cognitive Stimulation survey, with questions adapted from parent interview questions from the Early Childhood Longitudinal Studies program (Tourangeau et al., 2004), was implemented including questions measuring family-to-child reading, from which an HLE score was extracted: frequency with which a child is read to by a family member each week (“In a typical week, how often do you or any other family members read books to your child in English?”: not at all, once or twice a week, three to six times a week, and every day).Details on reading practices and demographics are displayed below (see Tables 1 and 2). Distributions of reading practices are included in the Supplementary Material (see Supplementary Figure S1; Supporting Information can be found at https://doi.org/10.1162/NOL.a.272).

**Table 1.** Reading practices.

**Covariate**	**Description**	**Median (IQR, range)**
Younger cohort		
Family-to-child reading	“How long per day is your child read to by an adult?” (0–15 min [1], 15–30 min [2], 30–60 min [3], more than 1 hr per day [4], more than 2 hr per day [5])	2 (0.5, 1–4)
Older cohort		
Family-to-child reading	“In a typical week, how often do you or any other family members read books to your child in English?” (not at all [1], once or twice a week [2], 3–6 times a week [3], every day [4])]	3 (2, 1–4)

*Note*. The table displays reading practices for the younger (5- to 6-year-olds) and older (7- to 8-year-olds) cohort. IQR = interquartile range.

**Table 2.** Demographics.

**Nuisance covariate**	**Description**	**Mean (*SD*, range)**	
		Younger cohort	Older cohort
Age	Average of participant age in months at time of DWI scan	70.9 (3.0, 66–81)	91.2 (4.5, 85–101)
		**Median (IQR, range)**	
Socioeconomic status	Highest grade/degree completed by mother with 1 = *high school*, 2 = *some college*, 3 = *associate degree*, 4 = *bachelor’s degree*, and 5 = *master’s degree or higher*	4 (2, 1–5)	4 (2, 1–5)

*Note*. The table displays demographic (age, socioeconomic status) information for the younger (5- to 6-year-olds) and older (7- to 8-year-olds) cohort. IQR: interquartile range; DWI = diffusion weighted imaging.

From the surveys administered, only questions on family-to-child reading were extracted for HLE analysis. The surveys included other questions on child independent reading. However, given that the nature of independent reading time can vary greatly for kids at this age and because some literature suggests there may be no meaningful beneficial effects without monitoring or feedback (Erbeli & Rice, 2022; Kelley & Clausen-Grace, 2010), we chose not to include this question in analyses in the present article. Using one question per age group to index HLE is a limitation of this study, as HLE could be characterized more fully with additional metrics that tap into the quality of the experience. Furthermore, HLE often also includes measures of access to reading-related resources. The present study focused on only shared reading practices, in line with prior literature on this age group that has found relations between shared reading and DWI (Davison et al., 2023; see Table 1).

#### Standardized assessments

Additionally, to assess participants’ nonverbal IQ and language skills, a series of standardized tests were administered. Nonverbal IQ was measured using the Kaufman Brief Intelligence Test, Second Edition (KBIT-2; Kaufman & Kaufman, 2004). The KBIT-2 nonverbal IQ score is composed of the Matrices subtest. This subtest incudes 42 items that use meaningful and abstract visual stimuli. The participant selects which of the images goes with the stimulus picture, completes the visual analogy, or solves a matrix of images. General language skill was measured using the Core Language Scale (CLS) score on the Clinical Evaluation of Language Fundamentals, Fifth Edition (CELF-5, Wiig et al., 2013). The CELF-5 CLS is considered a measure of general language ability, and for children 5–8 years old, this score is derived by summing the scaled scores from the CELF-5 subtests of sentence comprehension, word structure, formulated sentences, and recalling sentences. All children included in the study had normal IQ, as indexed by a standardized score of 80 or higher on the KBIT-2, and typical language abilities, as indexed by a standardized CLS score of 80 or higher on the CELF-5.

All children also completed standardized assessments of semantics, measured using the CELF-5 Word Classes subtest, and phonological awareness, measured using the Comprehensive Test of Phonological Processing (CTOPP-2) Elision subtest (Wagner et al., 2013). Scaled scores from these measures of phonology and semantics were used in analysis (see Table 3). Children who scored more than 3 standard deviations from the mean on the CELF-5 Word Classes or CTOPP-2 Elision were also identified as outliers and excluded from analysis.

**Table 3.** Standardized assessment performance.

**Assessment**	**Description**	**Mean (*SD*, range)**	
		Younger cohort	Older cohort
CTOPP Elision	A scaled score, based on age, for phonological awareness	11.5 (2.1, 6–18)	11.4 (2.6, 6–17)
		**Mean (*SD*, range)**	
CELF-5 Word Classes	A scaled score, based on age, for semantics	12.9 (3.3, 6–19)	12.5 (3.2, 3–19)

*Note*. The table displays scaled score information for the younger (5–6-year-olds) and older (7–8-year-olds) cohort.

CELF-5 Word Classes contains 40 items. For the first 12 items, the examiner shows the participant three or four images, says the names of the pictures out loud for the participant, and then asks the participant to tell them which two of those words go together in terms of their meaning. For example, for Item 2, the participant is told and shown the images of “Marker, Pencil, Strawberry” (the participant should choose Marker and Pencil). Starting with Item 13, the participant is told four words auditorily, with no pictures, and the participant must state which two of those words go together in terms of their meaning. For a 5- to 5.5-year-old, a scaled score of 10 means the participant got 11–12 items correct. For a 7-year-old, a scaled score of 10 means the participant got 20–21 items correct (see Table 2).

CTOPP-2 Elision contains 34 items. This subtest asks participants to remove a designated part from a spoken word to form a new word. For the first nine items, the examiner says a word and asks the examinee to say that word and then say that word after dropping a designated word part. For example, for Item 2, the participant is told “Say ‘cowgirl.’ Now say ‘cowgirl’ without saying ‘girl’” (the participant would say “cow”). For the next 25 items, the participant is told a word, they repeat that word, and then the examiner asks them to say that word without a particular sound. For example, Item 10 is when the participant is told “Say ‘cup.’ Now say ‘cup’ without saying /k/.” The correct response from the participant is “up.” For a 5- to 5.5-year-old, a scaled score of 10 means the participant got seven to nine items correct. For a 7- to 7.5-year-old, a scaled score of 10 means the participant got 19–21 items correct.

Participants were recruited in the Austin, Texas, metropolitan area. In the 5- to 6-year-old age group (*N* = 79, 48 girls), 23% identified as Hispanic or Latino. Eighty-five percent of participants identified as White, 5% identified as Black or African American, 1% identified as Asian, and 9% identified as multiracial. In the 7- to 8-year-old age group (*N* = 108, 68 girls), 21% identified as Hispanic or Latino. Eighty-two percent of participants identified as White, 4% identified as Black or African American, 1% identified as Asian, 1% identified as American Indian or Alaskan Native, and 12% identified as multiracial.

### DWI Data Acquisition

According to Wang et al. (2022), participants typically had two scan days, and the total scan duration for each day was approximately 1 hr. DWI data were acquired when there was extra time after children finished the fMRI tasks on that scan day. Experimenters visually examined data quality of each scan and repeated the bad scans if time allowed. Participants were invited for scanning on additional days if they had not finished the functional tasks. In the full data set for the 5- to 6-year-old age group, 10 of the 87 children repeated the DWI scan. In the 7- to 8-year-old age group, four of the 132 children repeated the DWI scan. If both DWI scans were fully complete, the one that needed fewer volumes removed due to slice-wise artifacts was used in analysis.

Participants were positioned supine in the MRI scanner, and foam pads were placed around the head to minimize movement. During DWI scans, participants watched a movie to increase comfort.

Images were acquired using a 3.0-T Siemens Skyra scanner with a 64-channel head coil. DWIs were collected using single-shot echo planar imaging with the following parameters: GRAPPA accel.factor PE = 2, TR = 5,000 ms, TE = 71 ms, field of view = 256 mm, matrix size = 128 × 128, bandwidth = 1502 Hz/Px, slice thickness = 2 mm, number of slices = 57, voxel size = 2 mm isotropic, flip angle = 90°, multiband acceleration factor = 3, b-value1 = 0 s/mm^2^, b-value2 = 800 s/mm^2^, echo spacing = 0.75 ms, diffusion directions = 64. Slices were acquired interleaved from foot to head.

### DWI Data Analysis

#### Preprocessing

The T1-weighted structural image was used to generate a brain mask by removing non-brain tissue using the Brain Extraction Tool from Functional Magnetic Resonance Imaging of the Brain (FMRIB) Software Library (Smith, 2002). Then, DWI Digital Imaging and Communications in Medicine (DICOM) data were converted into Nearly Raw Raster Data (NRRD; teem.sourceforge.net/nrrd/) format using DicomToNrrdConverter software from Slicer4 (www.slicer.org).

DWI data quality was assessed using DTIPrep (v1.2.8; Oguz et al., 2014). Quality check procedures included verification of image information, diffusion parameters, and automated artifact detection slice-wise correlation analysis. The slice-wise correlation analysis detects inter-slice brightness artifacts via normalized correlation between successive slices within a single DWI volume, and the results are reported in a quality control report for each participant (Oguz et al., 2014). Using these quality control reports, gradients with three or more slices flagged by slice-wise correlation analysis (correlation threshold > 3.5 *SD* from baseline) were excluded from further analysis. Remaining data proceeded to tensor estimation; preprocessing was managed via the dtiInit processing pipeline in the VISTASOFT package (MATLAB toolkit; https://github.com/vistalab/vistasoft) that incorporates motion and eddy-current correction.

In the 5- to 6-year-old age group, from the included scans, zero to nine volumes were removed per participant (average = 0.84, *SD* = 1.46). In the 7- to 8-year-old age group, from the included scans, zero to nine volumes were also removed (average = 0.81, *SD* = 1.42). Thus, across age groups, participants ranged from 0 to 13% (zero to nine) volumes removed. Of the 79 children 5–6 years old who were included, 43 had zero volumes removed, and of the 107 children 7–8 years old who were included, 67 had zero volumes removed.

The data were processed with mrDiffusion, which is a toolbox from the VISTALab (Stanford Vision and Imaging Science and Technology) diffusion MRI software suite (www.vistalab.com) that includes motion and Eddy current correction and tensor-fitting estimations (Rohde et al., 2004). Diffusion tensors were fitted using linear least squares, and eigenvalues from the diffusion tensor estimation were used to compute FA (Basser et al., 1994).

Finally, Automatic Fiber Quantification (AFQ) was completed with the AFQ (github.com/jyeatman/AFQ) software package (Yeatman, Dougherty, Myall, et al., 2012). Each fiber tract was sampled to 100 equidistant nodes. The FA value was computed at each node along the fiber. AFQ segments the whole brain fiber group into 20 white matter tracts that are defined in a white matter atlas (Wakana et al., 2007). The analyses here were focused on four left-hemispheric tracts: AF, SLF, IFOF, and ILF. Moreover, instead of computing mean diffusion parameters (for FA, axial diffusivity, and radial diffusivity), AFQ computes these diffusion parameters using a weighted sum of each fiber’s value at a given node. More specifically, a fiber is weighted based on its Mahalanobis distance from the core or mean location of the tract (Johnson et al., 2014), which improves detection power for group differences.

Given prior studies that have consistently found significant relations of both language and HLE to FA (Davison et al., 2023; Farah et al., 2020; Mathur et al., 2026; Yeatman, Dougherty, Ben-Shachar, et al., 2012), we chose to examine relations with FA in the present article to minimize the number of total analyses. Future studies could examine relations with mean diffusivity, radial diffusivity, and axial diffusivity to provide a fuller picture on the relation of HLE to white matter development, as some studies have shown relations to cognition for these metrics (Farah et al., 2020).

#### Nodes of interest

To establish nodes of the tract that were most associated with phonology or semantics, AFQ was used to compute values at the node level for correlation analysis. All analyses were done separately for each age group. To find nodes within each tract that were related to phonology, the correlation between CTOPP Elision and the FA value at each node of the left AF and left SLF was calculated. The FA values for the 30 nodes (minimum cluster size of three nodes) from each tract with the lowest *p* values from this correlation were extracted for analysis. These analyses were completed in MATLAB. This same process was completed for semantics by doing a correlation between CELF-5 Word Classes and the FA value of the left IFOF and left ILF. Nodes of interest (NOIs) were established separately for each age group. Given that FA changes to different degrees across different portions of the tract throughout development (Yeatman, Dougherty, Myall, et al., 2012), it is possible that FA of different nodes may be most related to phonology or semantics in different age groups. Some of the nodes in the NOIs overlap between groups, and nodes included in the masks are highlighted in gray, with overlapping nodes bolded in the Supplementary Material (see Supplementary Table S1), so that the overlapping nodes between age groups in the NOIs can be readily identified. Correlations with HLE were also completed across all 100 nodes of the tract for both age groups, allowing for fuller comparison.

#### HLE partial correlation analyses—primary planned analyses

The relation of HLE (controlling for SES and age) to the approximately 30 nodes identified based upon their correlation with phonology score (CTOPP-2 Elision Standardized Score) in the left AF and left SLF was examined. The relation of HLE (controlling for SES and age) to the 30 nodes identified based upon their correlation with semantics score (CELF-5 Word Classes Standardized Score) in the left IFOF and left ILF were also examined. Normality of the variables was tested with the Jarque–Bera test for normality. The results indicate that only CTOPP Elision and CELF Word Classes were normally distributed for both age groups; all the other variables (age, SES, HLE, and FA values) were not normally distributed, and thus, Spearman correlations were used. These analyses were completed in MATLAB, controlling for multiple comparisons as described below. Partial correlations, computed while controlling for age and SES, were used to examine relations between HLE and FA in line with prior literature that has examined these relations in similar age groups (Davison et al., 2023). We extended these prior analyses to examine relations in the nodes most associated with phonology or semantics.

To control for multiple comparisons, given the high degree of correlation between neighboring points on the tract, an implementation of the permutation method described by Nichols and Holmes and used in several studies (David et al., 2020; Yeatman, Dougherty, Myall, et al., 2012) was performed. The function AFQ_MultiCompCorrection randomly shuffled the data for 1,000 permutations, and a distribution of “chance” correlation values was created for each correlation of interest (e.g., HLE to FA of SLF controlling for age and SES in 5- to 6-year-olds). A final distribution from the maximum cluster size of these permutations was then created, and the actual (nonshuffled) cluster size was compared with these values to assign the significance alpha and cluster threshold at *p* < 0.05 (two-tailed). This procedure returned a family-wise error (FWE) corrected cluster size, which means that significant clusters of this size or greater do not need further *p*-value adjustment (Dodson et al., 2017; Dubner et al., 2020; Travis et al., 2015, 2016, 2017). Thus, primary planned comparisons are reported at a stringent threshold, requiring a sufficient number of adjacent nodes to meet the criteria for an FWE corrected cluster size. We considered this threshold to be strong evidence of a relation.

#### HLE partial correlation analyses—secondary planned analyses

Correlations were also completed with all 100 nodes of the tracts, rather than just the 30 nodes most correlated with phonology or semantics. Relations were examined at a corrected significance threshold, controlling for multiple comparisons with AFQ_MultiCompCorrection as described above.

Relations with HLE were examined not only at the FWE corrected cluster size but also at a more lenient threshold of *p* < 0.05 uncorrected and a cluster-wise threshold of 10% (three adjacent nodes for the 30 node mask and 10 adjacent nodes across the full tract of 100 nodes). Prior literature has examined a portion of the tract thought to be most relevant to the processes of interest (Dodson et al., 2017) and used a threshold of at least three adjacent nodes even when comparing across 100 nodes (Banfi et al., 2019). A prior study closely related to ours examined relations of at least three in 30 nodes (Mathur et al., 2026); thus, we chose three to be consistent with this related prior study. We considered this threshold to be weak evidence of a relation.

### Behavioral Analyses

For each age group, post hoc behavioral analyses were completed between HLE and standardized scores of phonology (CTOPP Elision) and semantics (CELF-5 Word Classes), controlling for age in months. Given the non-normal distributions of multiple variables (see the HLE Partial Correlation Analyses—Primary Planned Analyses section), Spearman partial correlations were completed. Significance was assessed at *p* < 0.0125 to account for the four tests conducted. There were no significant relations between HLE and phonology (*r* = −0.03, *p* = 0.74) or semantics (*r* = 0.09, *p* = 0.44) in the younger cohort nor HLE and phonology (*r* = −0.20, *p* = 0.04) or semantics (*r* = −0.06, *p* = 0.55) in the older cohort.

### Deviations From Preregistration

**Figure 1.**
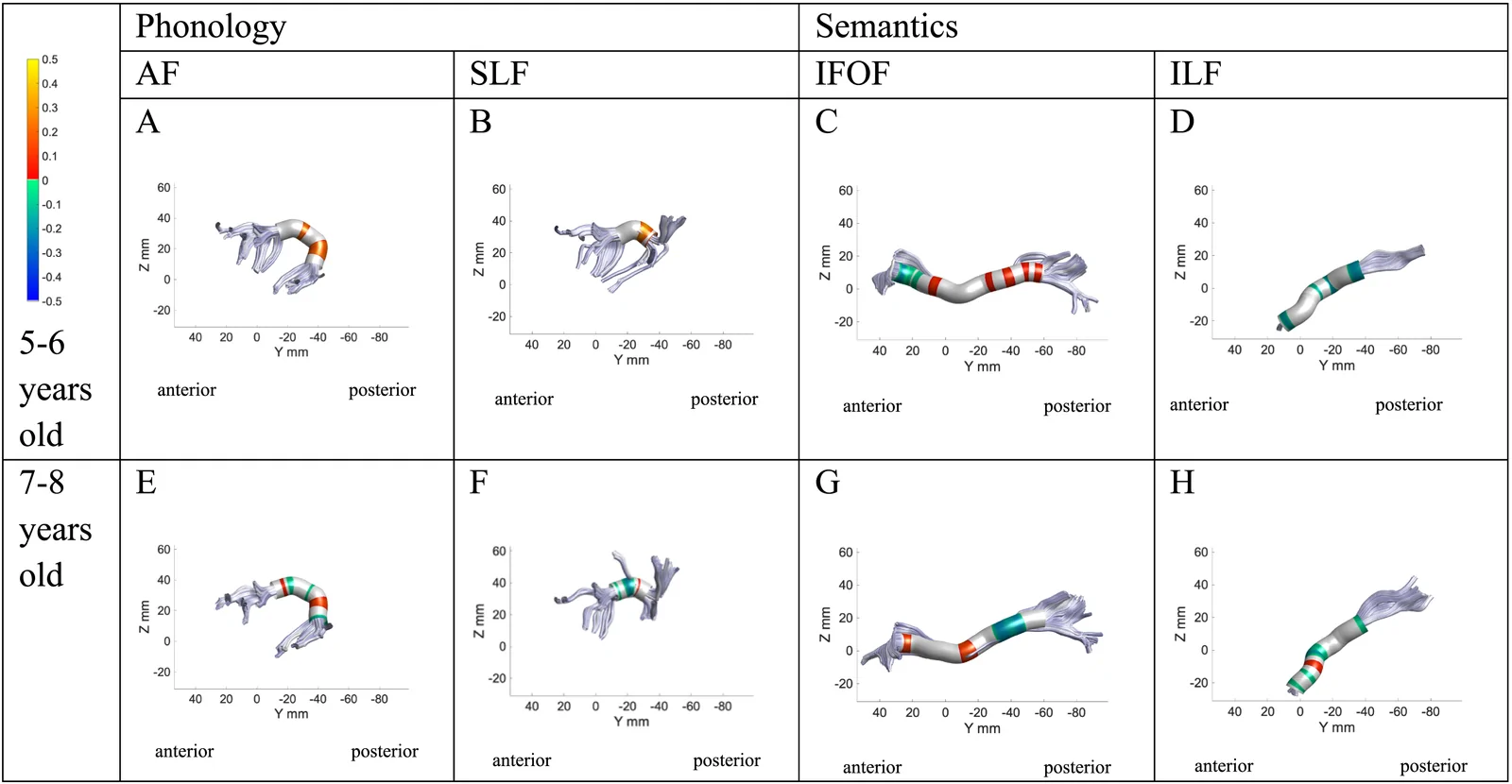
Masks of nodes most related to phonology or semantics. Correlation coefficients of the ∼30 most correlated nodes with phonology or semantics across tracts for each age group. Nodes are detailed in Supplementary Table S1. The group-level analyses are depicted on smoothed tracts from one participant. Supplementary Figure S2 shows full tractography from one participant. AF = arcuate fasciculus; SLF = superior longitudinal fasciculus; IFOF = inferior fronto-occipital fasciculus; ILF = inferior longitudinal fasciculus.

The first deviation from the preregistration is that 32 nodes were included for the IFOF mask in 5-year-olds and 33 nodes were included for the AF mask in 7-year-olds, rather than just 30 nodes. This decision was made because in some cases there were two nodes with a low *p* value that did not meet inclusionary criteria because the minimum cluster size we used was three nodes. However, when increasing the *p* value beyond that threshold, to get to 30 included nodes, a node adjacent to these two nodes then met inclusion criteria based upon the higher *p* value. This step created a cluster of three nodes, allowing more than 30 nodes to meet both the *p* value and cluster size threshold. Figure 1 depicts the isolated NOIs for each age group.

The second deviation is that for quality control, the preregistration states “DWI quality control (QC) procedures will be conducted using DTIprep software (Oguz et al., 2014) and visual inspection. Motion artifacts will be defined by translation threshold of 2.67 mm and rotation threshold of 0.5° through rigid registration-based volume-by-volume measures. Volumes with motion artifacts will be excluded from diffusion tensor estimation (a volume will be removed ≥3 bad slices).” For quality control in this study, we excluded data based on DTIPrep that were identified from interslice brightness artifacts via normalized correlation between successive slices. A volume with ≥3 bad slices was removed. Subsequently, we completed motion correction on all volumes with dtiInit form VISTASOFT. Correcting for motion, rather than removing volumes based on motion, is in line with other studies (Grotheer et al., 2022; Schurr et al., 2018; Yeatman, Dougherty, Myall, et al., 2012).

## RESULTS

### Preregistered Primary Analyses

In the NOIs, there were no significant clusters in regression analyses for either age group that would indicate strong evidence (corrected for multiple comparisons) of a positive correlation between HLE and FA, when controlling for age and SES. This was true for the NOIs identified by their correlation with phonology in the left AF and left SLF and for the NOIs identified by their correlation with semantics in the left IFOF and left ILF.

### Preregistered Secondary Analyses

**Figure 2.**
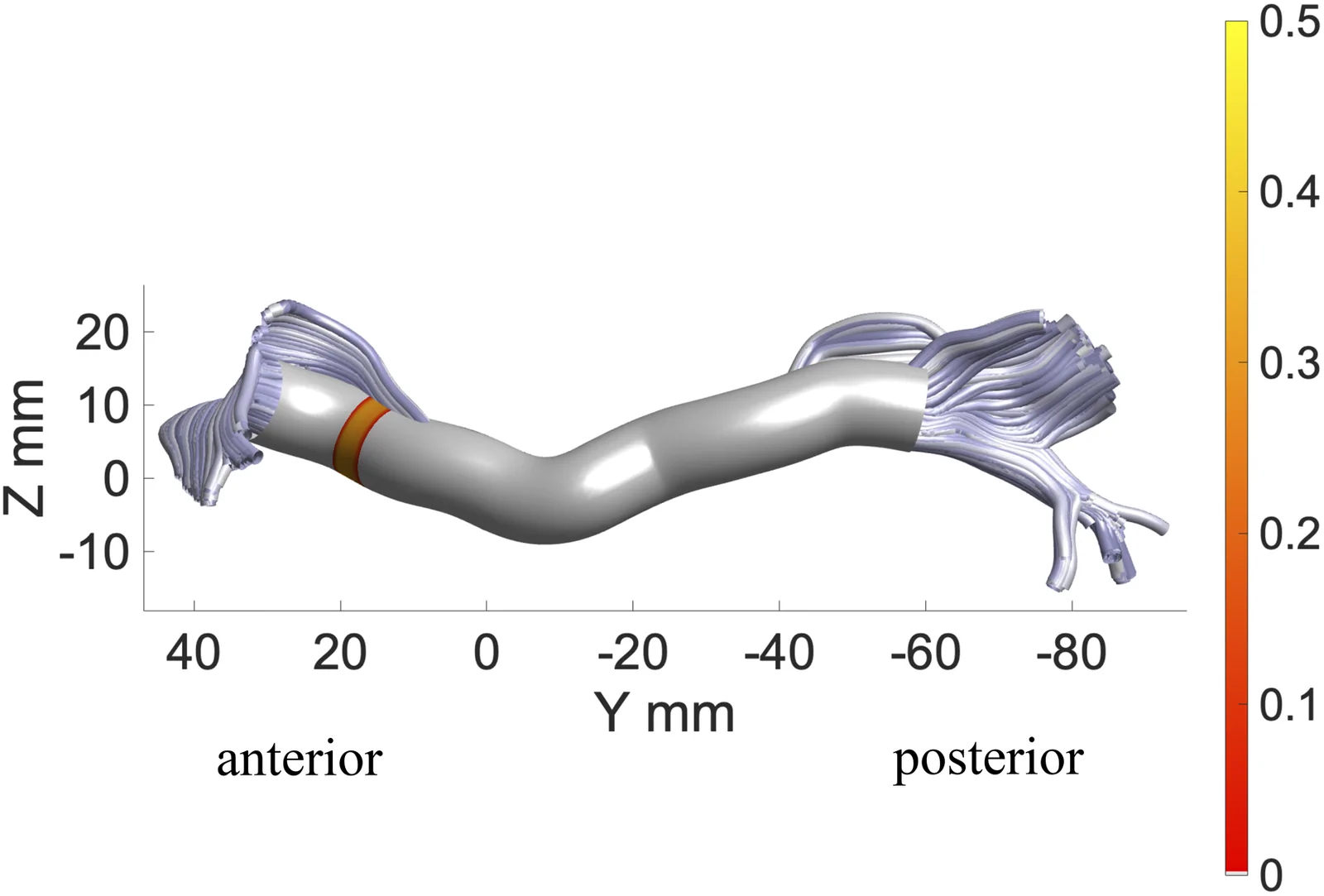
The relation of family-to-child reading to nodes in the left inferior longitudinal fasciculus in children 5–6 years old. Within ∼30 nodes most associated with semantics, controlling for age and socioeconomic status (*p* < 0.05 with a cluster-wise threshold of three nodes). The nodes identified are 81 (*r* = 0.2675), 82 (*r* = 0.2880), 83 (*r* = 0.3153), and 84 (*r* = 0.2877).

In the NOIs, we see weak evidence (*p* < 0.05 uncorrected and a cluster-wise threshold of three adjacent nodes) for a relation of HLE and FA, when controlling for age and SES, in the left IFOF in the younger group of 5- to 6-year-old children (see Figure 2). We did not find evidence for relations between HLE and FA in the other tracts nor in the older age group.

Across all 100 nodes of the tract, there was no strong nor weak evidence of a relation of HLE and FA in either age group. Table 4 depicts the null results that were found.

**Table 4.** The relation of family-to-child reading to tracts of interest.

	**Phonology**		**Semantics**	
	**AF**	**SLF**	**IFOF**	**ILF**
**5- to 6-year-olds**				
30 nodes	n.s.	n.s.	Weak evidence (see Figure 2)	n.s.
100 nodes	n.s.	n.s.	n.s.	n.s.
**7- to 8-year-olds**				
30 nodes	n.s.	n.s.	n.s.	n.s.
100 nodes	n.s.	n.s.	n.s.	n.s.

*Note*. Of the relations examined, only family-to-child reading and the left inferior fronto-occipital fasciculus in younger kids, in some of the 30 nodes most associated with semantics, demonstrated weak evidence of a relation; n.s. = not significant.

## DISCUSSION

Our study contributes to the literature in three main ways. First, it examines the relation of HLE to white matter integrity in relatively large groups of children ages 5–6 years old (*N* = 79) and 7–8 years old (*N* = 107), as no previous studies have investigated this relation in older children. Second, previous studies have examined relations in the AF and SLF, thought to be involved in phonology, and in the ILF, thought to be involved in semantics, but they have not investigated the IFOF, which has also been associated with semantics. Third, our study examines nodes that have been identified as related to phonology and semantics so that our conclusions have greater specificity. We only found weak evidence for a relation of HLE to white matter in the left IFOF in NOIs that had been identified as related to semantic knowledge.

The relation of parent–child reading to the left IFOF is in line with our prediction that we would see relations between HLE and language-related tracts. This relation was found in the 30 nodes most associated with semantic knowledge in the younger age group. The nodes associated with family-to-child reading in the left IFOF were located on the anterior portion of the tract. These nodes (81–84) were not a part of the mask defined in the left IFOF in older children. Prior work suggests that portions of the IFOF, extending to these anterior parts, may be involved in picture naming and visual semantic association (Giampiccolo et al., 2025). We may observe an association with HLE in younger children because books geared toward this age have more pictures than those geared toward older children. Prior literature suggests that picture book shared reading is common among young children, can vary in terms of its frequency and quality, and relates to language outcomes (Fletcher & Reese, 2005). In a randomized controlled trial of a shared reading intervention (*M*_age_ = 5.5), focused on teaching parents how to engage in dialogic reading and print referencing practices while reading, those in the intervention group had significantly more of an increase in skills including picture naming compared to children in the control group (Sim & Berthelsen, 2014). Thus, we may observe an association of HLE to the IFOF in younger children due to shared reading practices that involve picture books. In addition, the standardized assessment that we used to isolate the semantic NOIs was the CELF-5 Word Classes, which uses pictures for the first 12 items. A scaled score of 10 on this assessment for children 5–5.5 years old is 11–12 items, so for younger children, this test primarily involves semantic association and pictures. Therefore, the nodes identified in the left IFOF may be specifically related to picture naming, increasing the chances of detecting a relation with shared picture book reading in younger children.

At different ages, the books and the type of shared reading that takes place may differ. Picture book shared reading, for example, is common among young children (Fletcher & Reese, 2005). Flack et al. (2018) conducted a meta-analysis on the effects of shared storybook reading on word learning. The study found that storybooks read to older children include more target words and fewer occurrences of those words than those read to younger children, thus supporting more advanced vocabulary acquisition. Teacher resources also note that younger children (4–5 years old) often enjoy rhyming and concept books whereas older children (6–8 years old) may enjoy stories with more complex plot lines (Maddigan et al., 2005). Even if at 5 years of age, there is a link between HLE and nodes related to picture naming, at 7 years of age, HLE exposure may be capturing different constructs, like exposure to new key vocabulary words (Flack et al., 2018), so HLE at each timepoint may be linked to different nodes.

Davison et al. (2023) examined the relation of family-to-child reading to the AF, SLF, and ILF in children 4.8–6.2 years old, with the prediction that HLE would be especially associated with dorsal tracts given prior literature that suggests the AF and SLF may mature more slowly than ventral tracts (Brauer et al., 2013; Perani et al., 2011), and thus, that they could be more strongly influenced by environmental factors (Romeo et al., 2018). This previous study found a positive relation between family-to-child reading and FA of the left AF, as well as left lateralization of the SLF, but no relation with the ILF (Davison et al., 2023). A lateralization index was computed to indicate whether a tract in both hemispheres exhibited leftward or rightward lateralization by calculating the left average FA, subtracting the right average FA, and then dividing by the sum of these values and multiplying the final value by 100 (Davison et al., 2023). They also found that the relation between HLE and reading skill in second grade was mediated by the left lateralization of the SLF at kindergarten (Davison et al., 2023). Unlike this prior study, we did not find a relation of family-to-child reading to FA of the AF or SLF though we had a slightly larger sample of children in the present study. There are at least a couple of possible reasons for this discrepancy. Davison et al. (2023) do not report the exact language scores of the children in their study, but the authors note that around a third of children in their study exhibited phonological awareness composite, rapid naming composite, or letter sound knowledge standard scores below the 26th percentile. It could be that the relatively large percentage of participants with low phonologically related scores increased the sensitivity of their design to detect relations with the AF. In addition, our measure of family-to-child reading asked parents a multiple-choice question to select an interval for the amount or frequency of family-to-child reading. Davison et al. reports that they examined relations with the number of hours that someone spends reading to the child each week. The exact question is not listed, but if this is a free-response question or if more interval options are included, the additional specificity could have allowed for greater variance to detect a relation.

We predicted that there would be a relation between HLE and FA of the white matter tracts implicated in language processing. More specifically, we predicted that relations with tracts associated with phonology (i.e., AF, SLF) may be particularly strong in younger children and relations with tracts associated with semantics (i.e., IFOF, ILF) may be particularly strong in older children given the differences in the time course of the development of these skills (Ehri, 2020; Sénéchal & LeFevre, 2014) and potential age-related differences in the nature of the books read and shared reading practices (Maddigan et al., 2005). Prior work that examined the relation of family-to-child reading to brain activation in 5- to 6-year-old children and 7- to 8-year-old children found evidence of a relation to phonological processing in younger children and evidence of a relation to semantic processing in older children (Compton et al., 2026). Given the weak relation demonstrated in the current preregistered study with relatively large samples, future research is needed to investigate robust relations of shared reading to white matter structure. Particularly, studies with a large range of language skill and measures of not only quantity but also quality of HLE may further illuminate these relations. These characteristics would allow for a more thorough investigation given methodological differences with Davison et al. (2023).

When defining our masks, we isolated the nodes that were most associated with phonology (in the left AF or left SLF) or semantics (in the left IFOF and left ILF) for each age group. We chose the nodes with the lowest *p* values, regardless of the direction of the relation. Prior literature proposes that FA in particular tracts can increase or decrease as children age and develop particular skills because myelination, which increases FA, and pruning, which decreases FA, may both take place as the tract matures (Yeatman, Dougherty, Ben-Shachar, et al., 2012). Furthermore, the authors suggest that a variety of factors, including variation in the quality of early-life language input, may impact the rate at which these processes occur. Thus, even though FA mainly increases across most major white matter tracts as children develop, decreases in FA can occur in portions of the tract during points of maturation as pruning takes place (Yeatman, Dougherty, Ben-Shachar, et al., 2012). As illustrated in Figure 1 and Supplementary Table S1, the strongest relations with language skill appear to be positive for younger children between standardized phonology scores and FA in nodes of the left AF and left SLF. This is in line with prior literature in this age group that has demonstrated that higher phonological awareness is related to greater FA in the AF (Dodson et al., 2018; Saygin et al., 2013) and SLF (Dodson et al., 2018; Travis et al., 2017). Across other tracts, nodes that display negative relations do occur, but these tend to be weaker. For example, higher vocabulary knowledge (CELF-5 Word Classes scaled score) was related to less FA in a portion of the IFOF, with *r* values ranging from −0.0 to −0.1. In these nodes, we also showed that more family-to-child reading was associated with greater FA. These findings potentially indicate that at this age, strong relations between FA of the IFOF and semantics are not present, in line with some prior literature (Mathur et al., 2026; Vanderauwera et al., 2018; Vandermosten et al., 2015), but family-to-child reading does seem to relate to the FA of the tract and may support its development.

There are several limitations of the current study. Although the sample size for younger (*N* = 79) and older children (*N* = 107) is relatively large, the design was cross-sectional so different results for the age groups may be due to cohort effects. Other studies have reported a longitudinal link between reading and white matter development (Roy et al., 2024; Turesky et al., 2025) so longitudinal analyses that include examining the role of HLE would be important to understanding these relations. A second limitation is that we used one parent report question to index the amount or frequency of family-to-child reading, which does not indicate the quality of the shared book reading. Furthermore, these questions differed slightly for each age group due to differences in survey administration for each group. Future research would benefit from measures of HLE that ask more detailed questions about or directly measure reading practices. Future studies could also investigate additional components of HLE, such as availability of reading-related resources. A third limitation is that we used maternal education level as a measure of SES. Although educational attainment is commonly used as a proxy for SES, additional indicators such as occupational prestige or income can provide a more comprehensive characterization (Diemer et al., 2013). Another limitation is that the dissection of the tracts, which was done per person at the individual level using AFQ, may not capture the full width of tracts, including portions that may be relevant to processes of interest, as demonstrated by direct electrical stimulation studies (Giampiccolo & Duffau, 2022; Giampiccolo et al., 2025; Zemmoura et al., 2015). Example tractography from a participant is included in the Supplementary Material (see Supplementary Figure S2). This article was focused on tracts specifically related to phonology or semantics, but future research could also investigate relations with other tracts, including the frontal aslant tract that plays a specific and unique role in motor speech (Zhong et al., 2022).

Previous behavioral research has highlighted the importance of HLE to the development of phonological awareness and semantic knowledge (Niklas & Schneider, 2013; Sénéchal & LeFevre, 2014), and prior brain analyses have illustrated the importance of early shared reading (Horowitz-Kraus et al., 2024) and its relation to a limited number of white matter tracts in young children (Davison et al., 2023; Hutton et al., 2020; Turesky et al., 2022). We extended this research by examining several white matter tracts in younger as well as older children. We used a novel approach to identify nodes in dorsal tracts associated with phonology and ventral tracts associated with semantics. Our preregistered analytical plan found weak evidence for a relation of family-to-child reading with FA in the left IFOF at 5 years of age, suggesting that shared reading of picture books in younger children may be associated with the refinement of a tract that has been implicated in picture naming and semantic association. We did not find relations with other tracts, which is inconsistent with published work, highlighting the need for future research to demonstrate robust relations of HLE with brain metrics.

## FUNDING INFORMATION

James R. Booth, National Science Foundation (https://dx.doi.org/10.13039/100000001), Award ID: BCS-1358794. James R. Booth, National Institute on Deafness and Other Communication Disorders (https://dx.doi.org/10.13039/100000055), Award ID: R01-DC013274.

## AUTHOR CONTRIBUTIONS

**Alisha B. Compton**: Conceptualization; Formal analysis; Software; Visualization; Writing – original draft. **Avantika Mathur**: Conceptualization; Software; Writing – review & editing. **James R. Booth**: Conceptualization; Funding acquisition; Resources; Supervision, Writing – review & editing.

## DATA AND CODE AVAILABILITY STATEMENTS

The data set is publicly available on OpenNeuro.org (Wang et al., 2022; https://openneuro.org/datasets/ds003604). A copy of the exact codes used for this project can be found on GitHub (https://github.com/comptoab/HLE_PhonSem_WhiteMatter). The research questions, hypotheses, and analytical plan were preregistered through the Open Science Framework prior to beginning the data analyses (https://osf.io/9mhr8/).

## Supplementary Material



## TECHNICAL TERMS

Fractional anisotropy (FA): A metric of diffusion of water molecules that has been used to study white matter tracts.
Arcuate fasciculus (AF) and superior longitudinal fasciculus (SLF): Dorsal white matter tracts thought to be involved in phonological processing.
Phonology: The processing of sounds in language.
Inferior fronto-occipital fasciculus (IFOF) and inferior longitudinal fasciculus (ILF): Ventral white matter tracts pathways thought to be involved in semantic processing.
Semantics: The processing of meaning in language.
Home literacy environment (HLE): Reading-related resources and experiences in a child’s home.
Node-wise analysis: Dividing the tract into equidistant sections called nodes, given that white matter properties have been shown to vary across different portions of the tract.
Diffusion-weighted imaging (DWI): A type of magnetic resonance imaging scan that provides information on water molecule motion.
