# Supplementary material for: The Relation of the Home Literacy Environment to White Matter Pathways Associated with Language in Children 5–8 Years Old
Source: Neurobiol Lang (Camb). 2026 Jul 10;7:NOL.a.272. doi: 10.1162/NOL.a.272 (PMC13506227; doi:10.1162/NOL.a.272)
Supplement: Supplementary file 1 [file nol-07-272-s001.docx]

**Supplementary Material**

**Figure S1**

*Home Literacy Environment Distributions*

*Note.* Family-to-Child Reading responses for each group are displayed.

Table S1

Correlation coefficients of fractional anisotropy of nodes in relation to phonology or semantics

|  | 5-6-year-olds | | | | 7-8-year-olds | | | |
| --- | --- | --- | --- | --- | --- | --- | --- | --- |
|  | Phonology | | Semantics | | Phonology | | Semantics | |
| Node | AF | SLF | IFOF | ILF | AF | SLF | IFOF | ILF |
| 1 | 0.09 | -0.01 | -0.02 | **-0.16** | -0.01 | -0.01 | -0.06 | **-0.13** |
| 2 | 0.11 | 0.00 | 0.02 | **-0.16** | -0.02 | -0.01 | -0.07 | **-0.10** |
| 3 | 0.08 | 0.01 | 0.06 | **-0.19** | -0.02 | -0.01 | -0.07 | **-0.09** |
| 4 | 0.08 | 0.00 | 0.08 | **-0.20** | 0.00 | -0.02 | -0.07 | **-0.07** |
| 5 | 0.05 | 0.00 | 0.07 | **-0.19** | 0.01 | -0.03 | -0.06 | **-0.07** |
| 6 | 0.03 | 0.00 | 0.02 | -0.21 | 0.02 | -0.03 | -0.05 | -0.06 |
| 7 | 0.00 | 0.00 | -0.02 | -0.21 | 0.03 | -0.04 | -0.04 | -0.03 |
| 8 | -0.04 | 0.00 | 0.01 | -0.19 | 0.04 | -0.05 | -0.03 | 0.00 |
| 9 | -0.07 | -0.01 | 0.05 | -0.20 | 0.04 | -0.06 | 0.00 | 0.01 |
| 10 | -0.08 | -0.01 | 0.07 | -0.16 | 0.06 | -0.06 | 0.05 | 0.01 |
| 11 | -0.12 | -0.01 | 0.07 | -0.14 | 0.06 | -0.06 | 0.04 | 0.01 |
| 12 | -0.14 | -0.01 | 0.05 | -0.13 | 0.07 | -0.06 | 0.01 | -0.02 |
| 13 | -0.14 | 0.00 | 0.03 | -0.13 | 0.08 | -0.06 | -0.02 | -0.03 |
| 14 | -0.14 | -0.01 | -0.01 | -0.13 | 0.07 | -0.07 | -0.08 | -0.03 |
| 15 | -0.14 | 0.00 | -0.03 | -0.12 | 0.07 | -0.06 | -0.13 | -0.04 |
| 16 | -0.11 | 0.00 | -0.05 | -0.10 | 0.07 | -0.05 | -0.18 | -0.04 |
| 17 | -0.09 | 0.01 | -0.04 | -0.08 | 0.03 | -0.05 | -0.21 | -0.05 |
| 18 | -0.05 | 0.03 | -0.05 | -0.10 | 0.00 | -0.04 | -0.22 | -0.05 |
| 19 | -0.03 | 0.04 | -0.01 | -0.08 | -0.02 | -0.03 | -0.21 | -0.03 |
| 20 | 0.00 | 0.04 | 0.04 | -0.07 | -0.05 | -0.03 | -0.19 | -0.03 |
| 21 | -0.01 | 0.03 | **0.07** | -0.05 | -0.08 | -0.03 | **-0.16** | -0.02 |
| 22 | 0.00 | 0.02 | **0.10** | -0.05 | -0.10 | -0.03 | **-0.15** | 0.00 |
| 23 | 0.00 | 0.00 | **0.11** | -0.07 | -0.10 | -0.03 | **-0.14** | 0.01 |
| 24 | 0.02 | 0.00 | **0.08** | -0.08 | -0.09 | -0.02 | **-0.15** | 0.04 |
| 25 | 0.04 | -0.01 | 0.04 | -0.09 | -0.06 | -0.02 | -0.17 | 0.04 |
| 26 | 0.05 | -0.01 | 0.01 | -0.13 | -0.04 | -0.01 | -0.21 | 0.04 |
| 27 | 0.08 | 0.00 | 0.00 | -0.10 | -0.03 | 0.00 | -0.22 | 0.02 |
| 28 | 0.09 | -0.01 | 0.00 | -0.08 | -0.01 | -0.01 | -0.22 | 0.01 |
| 29 | 0.11 | -0.01 | 0.02 | -0.07 | 0.01 | -0.01 | -0.22 | 0.00 |
| 30 | 0.12 | -0.01 | 0.04 | -0.08 | 0.02 | 0.00 | -0.19 | -0.02 |
| 31 | 0.13 | 0.00 | 0.05 | -0.09 | 0.03 | -0.01 | -0.15 | -0.04 |
| 32 | 0.14 | 0.00 | 0.08 | -0.11 | 0.04 | -0.02 | -0.11 | -0.03 |
| 33 | 0.17 | 0.00 | 0.06 | -0.15 | 0.04 | -0.03 | -0.07 | -0.02 |
| 34 | 0.21 | 0.00 | 0.05 | -0.17 | 0.04 | -0.04 | -0.03 | -0.01 |
| 35 | 0.22 | -0.01 | 0.03 | -0.20 | 0.04 | -0.06 | -0.02 | 0.01 |
| 36 | 0.23 | -0.01 | 0.02 | -0.22 | 0.04 | -0.08 | -0.01 | 0.01 |
| 37 | 0.21 | -0.03 | 0.03 | -0.20 | 0.01 | -0.11 | -0.01 | -0.01 |
| 38 | 0.19 | -0.02 | 0.05 | -0.19 | -0.01 | -0.12 | -0.03 | -0.02 |
| 39 | 0.16 | -0.04 | 0.07 | -0.16 | -0.04 | -0.12 | -0.01 | 0.00 |
| 40 | 0.14 | -0.03 | 0.05 | -0.14 | -0.04 | -0.14 | 0.01 | -0.01 |
| 41 | 0.13 | -0.03 | -0.01 | -0.10 | -0.05 | -0.15 | 0.05 | 0.00 |
| 42 | 0.12 | -0.04 | -0.03 | -0.06 | -0.07 | -0.16 | 0.09 | 0.01 |
| 43 | 0.12 | -0.05 | -0.03 | -0.06 | -0.06 | -0.17 | 0.11 | 0.04 |
| 44 | 0.09 | -0.04 | -0.02 | -0.06 | -0.06 | -0.19 | 0.11 | 0.05 |
| 45 | 0.08 | -0.05 | 0.02 | -0.05 | -0.06 | -0.20 | 0.10 | 0.07 |
| 46 | 0.09 | -0.04 | 0.04 | -0.06 | -0.06 | -0.21 | 0.09 | 0.07 |
| 47 | 0.07 | -0.03 | 0.06 | -0.07 | -0.06 | -0.20 | 0.06 | 0.07 |
| 48 | 0.06 | -0.01 | 0.06 | -0.09 | -0.07 | -0.19 | 0.05 | 0.04 |
| 49 | 0.05 | 0.00 | 0.03 | -0.09 | -0.08 | -0.17 | 0.07 | -0.02 |
| 50 | 0.05 | 0.02 | 0.00 | -0.10 | -0.09 | -0.17 | 0.11 | -0.06 |
| 51 | 0.04 | 0.03 | -0.04 | -0.13 | -0.08 | -0.15 | 0.13 | -0.08 |
| 52 | 0.03 | 0.05 | -0.05 | **-0.14** | -0.08 | -0.13 | 0.14 | **-0.09** |
| 53 | 0.02 | 0.06 | -0.05 | **-0.14** | -0.06 | -0.09 | 0.14 | **-0.08** |
| 54 | 0.02 | 0.08 | -0.03 | **-0.15** | -0.05 | -0.07 | 0.13 | **-0.08** |
| 55 | 0.03 | 0.10 | 0.02 | **-0.15** | -0.04 | -0.04 | 0.13 | **-0.08** |
| 56 | 0.04 | 0.12 | 0.01 | **-0.14** | -0.03 | -0.02 | 0.15 | **-0.09** |
| 57 | 0.05 | 0.15 | -0.01 | -0.13 | -0.03 | 0.00 | 0.11 | -0.12 |
| 58 | 0.06 | 0.18 | -0.05 | -0.11 | -0.03 | 0.02 | 0.08 | -0.14 |
| 59 | 0.07 | 0.20 | -0.05 | -0.08 | -0.02 | 0.02 | 0.06 | -0.17 |
| 60 | 0.08 | 0.23 | -0.04 | -0.01 | -0.01 | 0.03 | 0.03 | -0.19 |
| 61 | 0.08 | 0.25 | -0.04 | 0.02 | 0.02 | 0.04 | -0.01 | -0.20 |
| 62 | 0.09 | 0.27 | -0.04 | 0.02 | 0.04 | 0.05 | -0.04 | -0.17 |
| 63 | 0.09 | **0.29** | -0.03 | -0.02 | 0.06 | **0.06** | -0.04 | -0.13 |
| 64 | 0.12 | **0.30** | 0.01 | -0.02 | 0.08 | **0.06** | -0.02 | -0.07 |
| 65 | 0.14 | **0.30** | 0.02 | -0.02 | 0.06 | **0.05** | 0.00 | -0.03 |
| 66 | **0.17** | 0.31 | 0.03 | -0.02 | **0.08** | 0.05 | -0.02 | -0.01 |
| 67 | **0.18** | 0.31 | 0.03 | -0.03 | **0.09** | 0.05 | -0.05 | 0.03 |
| 68 | **0.19** | 0.29 | 0.04 | -0.03 | **0.10** | 0.04 | -0.05 | 0.03 |
| 69 | **0.20** | 0.30 | 0.06 | -0.03 | **0.12** | 0.04 | -0.04 | 0.04 |
| 70 | **0.20** | 0.29 | 0.09 | -0.05 | **0.14** | 0.04 | -0.01 | 0.06 |
| 71 | **0.20** | 0.28 | 0.12 | -0.08 | **0.16** | 0.04 | 0.02 | 0.07 |
| 72 | **0.19** | 0.26 | 0.08 | -0.05 | **0.14** | 0.04 | 0.05 | 0.08 |
| 73 | **0.19** | 0.25 | 0.04 | -0.04 | **0.13** | 0.04 | 0.04 | 0.09 |
| 74 | **0.19** | 0.23 | 0.02 | -0.03 | **0.12** | 0.04 | 0.01 | 0.08 |
| 75 | **0.16** | 0.23 | 0.01 | -0.03 | **0.13** | 0.05 | 0.00 | 0.07 |
| 76 | **0.17** | 0.21 | -0.01 | -0.02 | **0.13** | 0.05 | 0.01 | 0.05 |
| 77 | **0.17** | 0.20 | 0.01 | 0.02 | **0.12** | 0.04 | -0.01 | 0.03 |
| 78 | **0.18** | 0.19 | 0.00 | 0.04 | **0.10** | 0.04 | 0.01 | -0.01 |
| 79 | **0.18** | 0.19 | 0.00 | 0.01 | **0.08** | 0.04 | 0.04 | -0.03 |
| 80 | 0.19 | 0.17 | 0.01 | -0.02 | 0.06 | 0.03 | -0.02 | -0.03 |
| 81 | 0.19 | 0.17 | -0.10 | -0.06 | 0.03 | 0.02 | -0.02 | -0.05 |
| 82 | 0.19 | 0.16 | -0.10 | -0.06 | 0.02 | 0.01 | -0.03 | -0.09 |
| 83 | 0.21 | 0.15 | -0.09 | -0.06 | 0.02 | 0.01 | -0.04 | -0.11 |
| 84 | 0.22 | 0.15 | -0.07 | -0.07 | 0.02 | 0.01 | -0.04 | -0.08 |
| 85 | 0.21 | 0.14 | 0.01 | -0.04 | 0.03 | 0.01 | -0.05 | -0.06 |
| 86 | 0.20 | 0.14 | 0.00 | -0.05 | 0.02 | 0.01 | -0.04 | -0.05 |
| 87 | 0.17 | 0.14 | -0.03 | -0.04 | 0.02 | 0.00 | -0.03 | -0.01 |
| 88 | 0.13 | 0.13 | -0.06 | -0.06 | 0.00 | -0.01 | -0.02 | 0.00 |
| 89 | 0.09 | 0.12 | -0.09 | -0.09 | -0.02 | -0.01 | 0.02 | 0.00 |
| 90 | 0.07 | 0.11 | -0.09 | -0.10 | -0.02 | -0.02 | 0.06 | 0.00 |
| 91 | 0.05 | 0.10 | -0.08 | -0.13 | 0.00 | -0.02 | 0.09 | -0.01 |
| 92 | 0.02 | 0.11 | -0.05 | -0.15 | -0.01 | -0.03 | 0.12 | -0.02 |
| 93 | 0.01 | 0.11 | **-0.07** | -0.15 | -0.03 | -0.03 | **0.14** | -0.03 |
| 94 | 0.00 | 0.12 | **-0.14** | -0.18 | -0.07 | -0.03 | **0.12** | -0.04 |
| 95 | -0.01 | 0.12 | **-0.20** | **-0.20** | -0.11 | -0.03 | **0.14** | **-0.08** |
| 96 | -0.05 | 0.12 | **-0.21** | **-0.17** | -0.13 | -0.03 | **0.13** | **-0.09** |
| 97 | -0.08 | 0.14 | **-0.21** | **-0.14** | -0.14 | -0.02 | **0.12** | **-0.08** |
| 98 | -0.08 | 0.16 | -0.18 | -0.10 | -0.14 | -0.01 | 0.05 | -0.04 |
| 99 | -0.12 | 0.16 | -0.08 | -0.07 | -0.15 | -0.01 | -0.01 | -0.06 |
| 100 | -0.15 | 0.16 | -0.05 | -0.04 | -0.16 | -0.01 | -0.06 | -0.09 |
| *Average* | *0.0744* | *0.0869* | *-0.0031* | *-0.0964* | *0.0025* | *-0.0326* | *-0.0139* | *-0.0273* |

*Note.* Clusters of ~30 most related FA of white matter nodes to phonology or semantic standardized scores. The nodes in gray are included in the masks, and the bolded nodes overlap for that tract between age groups.

**Figure S2**

*Tractography from Example Participant*

*Note.* Tracts from one participant are shown in full.
